# Comparison of Apoptosis and Autophagy in Human Chondrocytes Induced by the T-2 and HT-2 Toxins

**DOI:** 10.3390/toxins11050260

**Published:** 2019-05-08

**Authors:** Fang-Fang Yu, Xia-Lu Lin, Xi Wang, Zhi-Guang Ping, Xiong Guo

**Affiliations:** 1 Department of Epidemiology and Biostatistics, College of Public Health, Zhengzhou University, Zhengzhou 45001, China; yufangfang@zzu.edu.cn (F.-F.Y.); pingzhg@zzu.edu.cn (Z.-G.P.); 2 NHC Key Laboratory of Trace Elements and Endemic Diseases, Institute of Endemic Diseases, School of Public Health of Health Science Center, Xi’an Jiaotong University, Xi’an 710061, China; summer2047@stu.xjtu.edu.cn (X.-L.L.); wn18andlife@xjtu.edu.cn (X.W.)

**Keywords:** T-2 toxin, HT-2 toxin, apoptosis, autophagy

## Abstract

In this report, we have investigated the apoptosis and autophagy of chondrocytes induced by the T-2 and HT-2 toxins. The viability of chondrocytes was measured by the MTT assay. Malondialdehyde (MDA) and superoxide dismutase (SOD) kits were used to measure the oxidative stress of chondrocytes. The apoptosis of chondrocytes was measured using flow cytometry. Hoechst 33258 and MDC staining agents were introduced to analyze apoptosis and autophagy induction in chondrocytes, respectively. Protein expression of Bax, caspase-9, caspase-3, and Beclin1 was examined by western blotting analysis. The T-2 and HT-2 toxins significantly decreased the viability of chondrocytes in a time-dependent manner. The level of oxidative stress in chondrocytes induced by the T-2 toxin was significantly higher when compared with that of the HT-2 toxin. The apoptosis rate of chondrocytes induced by the T-2 toxin increased from 3.26 ± 1.03%, 18.38 ± 1.28%, 34.5 ± 1.40% to 49.67 ± 5.31%, whereas apoptosis rate of chondrocytes induced by the HT-2 toxin increased from 3.82 ± 1.03%, 11.61 ± 1.27%, 25.72 ± 2.95% to 36.28 ± 2.81% in 48 h incubation time. Hoechst 33258 staining confirmed that apoptosis of chondrocytes induced by the T-2 toxin was significantly higher than that observed when the chondrocytes were incubated with the HT-2 toxin. MDC staining revealed that the autophagy rate of chondrocytes induced by the T-2 toxin increased from 6.38% to 63.02%, whereas this rate induced by the HT-2 toxin changed from 6.08% to 53.33%. The expression levels of apoptosis and autophagy related proteins, Bax, caspase-9, caspase-3, and Beclin1 in chondrocytes induced by the T-2 toxin were significantly higher when compared with those levels induced by the HT-2 toxin.

## 1. Introduction

Kashin–Beck disease (KBD) is an endemic, chronic, and deformed osteoarthropathic disease. There are 0.64 million KBD patients distributed from northeast to southwest regions of China. KBD patients suffer from joint pain, morning stiffness, limited motion, and joint enlargement [1,2]. Three underlying risk factors are considered to be responsible for KBD: mycotoxin (T-2 toxin) in grain, selenium deficiency, and organic acid in drinking water [3,4]. Recently, research effort has focused on apoptosis of human chondrocytes induced by the T-2 toxin [5]. Previous epidemiologic studies have confirmed the presence of higher concentrations of the T-2 toxin in grains from endemic areas [6]. The T-2 toxin has been showed to exert various toxin effects on experimental animals and human chondrocytes, including the dysplasia tibial growth plate cartilage in chicken, and induction of apoptosis of human chondrocytes, which involves p53, Bcl-xL, Bcl-2, Bax, and caspase-3 signaling pathways [7].

T-2 toxin is hydrolyzed to the HT-2 toxin in nature. For example, the T-2 toxin is rapidly metabolized to the HT-2 toxin in microsomes of liver, kidney, and spleen with conversion rates of 80%. The T-2 toxin detected in grains can be rapidly converted in vivo to the HT-2 toxin after consuming the contaminated food. The T-2 toxin rapidly combines with proteins in the blood and is delivered to organs through the mouth, skin, and respiratory tract [8]. The T-2 toxin is metabolized to the HT-2 toxin in the liver after entering enterohepatic circulation. Rats treated with T-2 toxin for 8 h, were found to convert the toxin at different rates in various tissues with conversion rates ranging from 68.20% to 90.70%, and the T-2 and HT-2 toxins were both detected in the skeletal system (thighbone, knee joint, and costal cartilage) [9]. The T-2 toxin in cultured chondrocytes is metabolized into the HT-2 toxin. The concentration of the T-2 toxin in the cell medium was found to decrease from 20 to 6.67 ng/mL during 48 h incubation period, while the concentration of the HT-2 toxin increased from 0 to 6.88 ng/mL over the same period [10]. Metabolism of the T-2 toxin to the HT-2 toxin in the liver and digestive systems can directly affect the human skeletal system. However, the toxic effects of the HT-2 toxin on human chondrocytes remain poorly understood.

In this study, human chondrocytes were cultured in fetal bovine serum (FBS) media. The chondrocytes were exposed to the T-2 and HT-2 toxins for 48 h, and the resulting apoptotic and autophagic effects were monitored.

## 2. Results

### 2.1. Viability of Chondrocytes Induced by the T-2 and HT-2 Toxins

**Figure 1:**
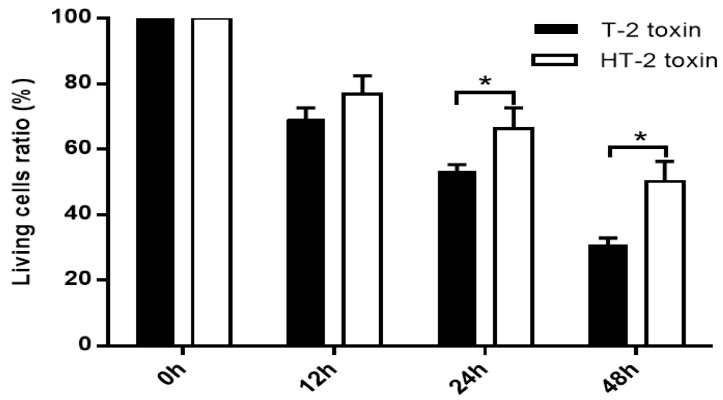
Effects of same concentration of the T-2 and HT-2 toxins on the cellular viability of chondrocytes were estimated by MTT reduction. * *p* < 0.05 was considered as significant difference between the two groups.

Chondrocytes were incubated with the T-2 and HT-2 toxins (20 ng/mL) for 48 h. As shown in Figure 1, the T-2 and HT-2 toxins decreased the viability of chondrocytes significantly in a time-dependent manner, and the toxic effect of the T-2 toxin on the viability of chondrocytes was significantly higher than that of the HT-2 toxin after 24 h and 48 h incubation time. Therefore, the toxic effect of the T-2 toxin on the viability of chondrocytes was significantly higher when compared with that of the HT-2 toxin.

### 2.2. Oxidative Stress of Chondrocytes Induced by the T-2 and HT-2 Toxins

**Figure 2:**
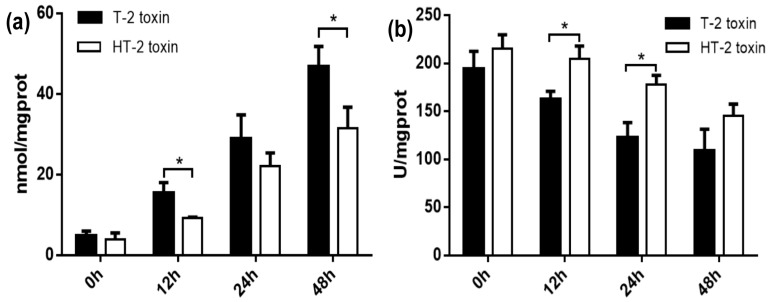
The malondialdehyde (MDA) (**a**) and superoxide dismutase (SOD) (**b**) content in the chondrocytes were induced by the T-2 and HT-2 toxins. * *p* < 0.05 was considered as significant difference between the two groups.

MDA is an important indicator of lipid peroxidation damage in tissue and cells. As shown in Figure 2a, the chondrocytes were treated with the T-2 and HT-2 toxins (20 ng/mL) for 48 h. The MDA content in the chondrocytes increased as the incubation time in the presence of the two toxins increased. At the same dose and incubation period (12 h and 48 h), the MDA content in the chondrocytes induced by the T-2 toxin was significantly higher than the chondrocytes exposed to HT-2 toxin (*p* < 0.05).

SOD is an important antioxidant defense enzyme in humans. As shown in Figure 2b, the SOD content in the chondrocytes decreased as the incubation period of the T-2 and HT-2 toxins increased. At the same dose and incubation period (12 h and 24 h), the SOD content in the chondrocytes incubated with the T-2 toxin was significantly lower when compared with the SOD content present in chondrocytes cultured with the HT-2 toxin (*p* < 0.05).

### 2.3. Apoptosis of Chondrocytes Induced by the T-2 and HT-2 Toxins

**Figure 3:**
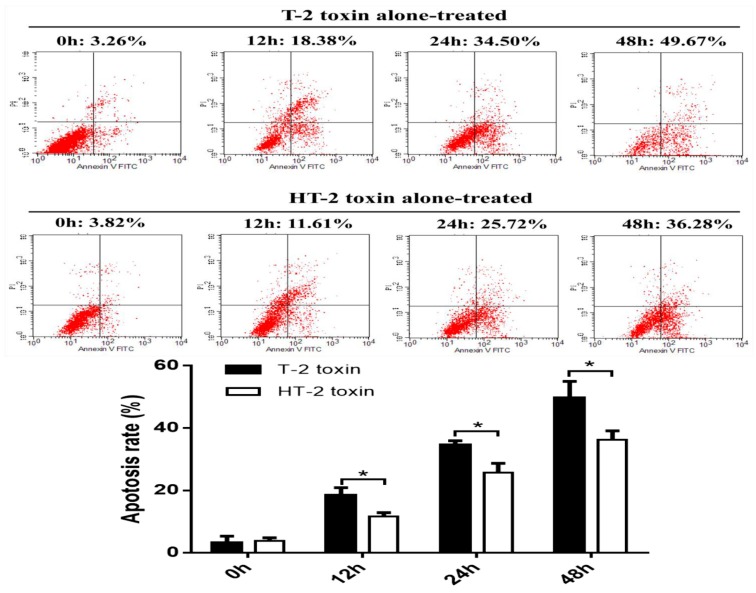
Apoptosis of chondrocytes was induced by the T-2 and HT-2 toxins using flow cytometry analysis. * *p* < 0.05 was considered as significant difference between two groups.

Flow cytometry was used to analyze apoptosis of chondrocytes induced by the T-2 and HT-2 toxins (20 ng/mL). Apoptosis of chondrocytes increased gradually as the incubation period with the toxins increased (Figure 3); the apoptosis of chondrocytes induced by the T-2 toxin increased in the range 3.26 ± 1.03%, 18.38 ± 1.28%, 34.5 ± 1.40%, and 49.67 ± 5.31% after incubation for 0, 12, 24, and 48 h, respectively, whereas apoptosis of chondrocytes induced by HT-2 toxin increased in the range 3.82 ± 1.03%, 11.61 ± 1.27%, 25.72 ± 2.95%, and 36.28 ± 2.81% over the same time. At the same dose and incubation period, the apoptosis of chondrocytes induced by the T-2 toxin was significantly higher than that induced by the HT-2 toxin, and the difference was statistically significant (*p* < 0.05).

**Figure 4:**
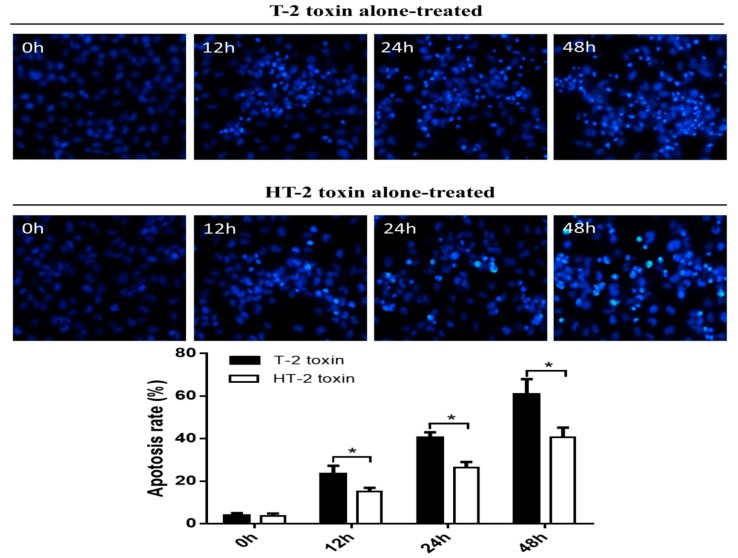
Apoptosis of chondrocytes was induced by the T-2 and HT-2 toxins using Hoechst 33258 staining. The cell nucleus with a white, thick dense cells were considered to be positive apoptosis cells under the fluorescence microscope (×400). * *p* < 0.05 was considered as significant difference between two groups.

Hoechst 33258 staining was used to analyze apoptosis of chondrocytes induced by the T-2 and HT-2 toxins. Cell nuclei that stained white and thick dense were considered to be positive apoptosis cells. As shown in Figure 4, the apoptosis rate of chondrocytes incubated with the T-2 toxin increased from 3.94% to 60.67%, whereas the apoptosis rate of chondrocytes incubated with the HT-2 toxin increased from 3.74% to 40.75%. Therefore, the apoptosis of chondrocytes induced by the T-2 toxin was significantly higher when compared with that of HT-2 toxin, and the difference was statistically significant between two groups (*p* < 0.05).

### 2.4. Apoptosis-Related Proteins in Chondrocyte Induced by T-2 and HT-2 Toxins

**Figure 5:**
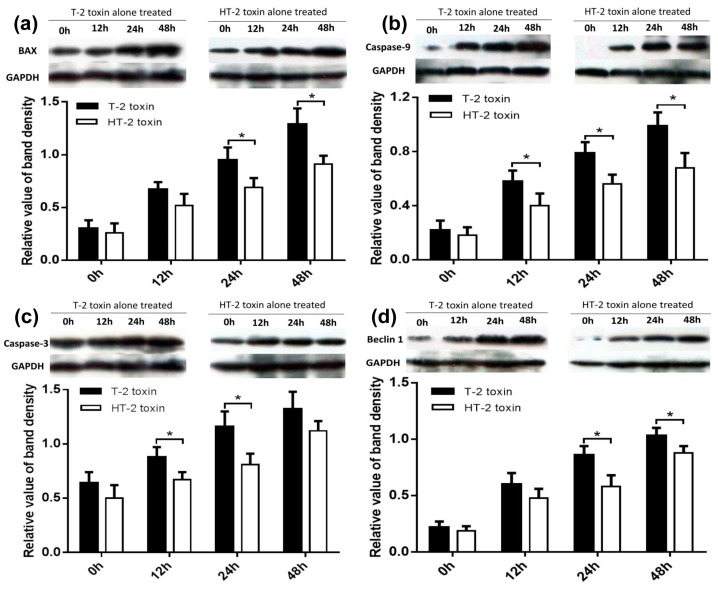
Apoptosis and autophagy related with proteins (**a**) Bax, (**b**) caspase-9, (**c**) caspase-3, and (**d**) Beclin1 of chondrocytes were induced by the T-2 and HT-2 toxins. The expression levels of Bax, caspase-9, caspase-3, and Beclin1 referred to the GAPDH (load control) were calculated in the T-2 toxin group and HT-2 toxin group. And then the expression levels of Bax, caspase-9, caspase-3, and Beclin1 were compared between two groups. * *p* < 0.05 was considered as significant difference between two groups.

As shown in Figure 5, chondrocytes incubated with the T-2 and HT-2 toxin (20 ng/mL) for 48 h shown an increased expression level of Bax (Figure 5a), caspase-9 (Figure 5b), and caspase-3 (Figure 5c), and the increases in protein levels were dependent on the incubation period. The relative expression level of Bax, caspase-9, and caspase-3 proteins in chondrocytes induced by the T-2 toxin was 1.29 fold, 0.99 fold, and 1.32 fold, respectively. The relative expression level of Bax, caspase-9, and caspase-3 proteins in chondrocytes induced by the HT-2 toxin was 0.91 fold, 0.68 fold, and 1.12 fold, respectively. The increased expression levels of Bax, caspase-9, and caspase-3 in chondrocytes induced by the T-2 toxin were statistically significant when compared with that of the HT-2 toxin (*p* < 0.05).

### 2.5. Autophagy of Chondrocytes Induced by the T-2 and HT-2 Toxins

**Figure 6:**
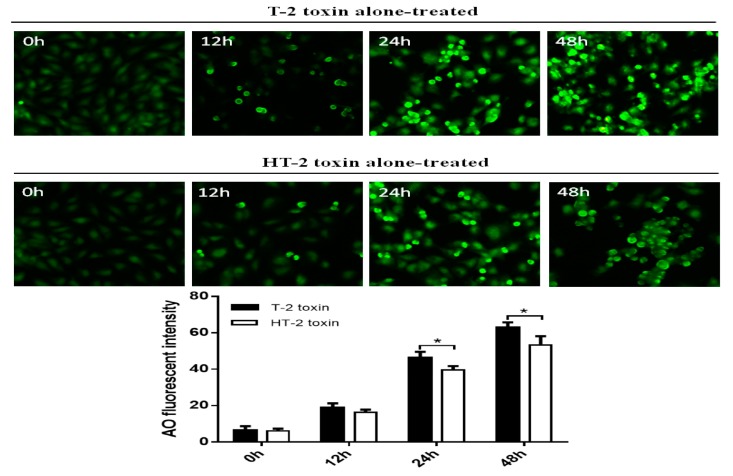
Autophagy of chondrocytes was induced by the T-2 and HT-2 toxins using MDC staining, cell nuclei stained cyan-green were positive for an acidic autophagosome under the fluorescence microscope (×400). * *p* < 0.05 was considered as significant difference between two groups.

As shown in Figure 6, the MDC kit was used to analyze autophagy of chondrocytes induced by the T-2 and HT-2 toxins (20 ng/mL). Cell nuclei stained cyan-green were positive for an acidic autophagosome. The autophagy rate of chondrocytes induced by the T-2 toxin increased from 6.38% to 63.02%, and the autophagy rate of chondrocytes induced by the HT-2 toxin increased from 6.08% to 53.33%. Therefore, the autophagy rate of chondrocytes induced by the T-2 toxin was significantly higher than that caused by the HT-2 toxin, and the difference was statistically significant (*p* < 0.05).

### 2.6. Autophagy-Related Proteins in Chondrocytes Induced by the T-2 and HT-2 Toxins

As shown in Figure 5d, the relative expression level of Beclin1 was observed to increase gradually as the incubation period increased. The relative expression level of Beclin1 in chondrocytes induced by the T-2 toxin was increased 1.03 fold, and the relative expression level of Beclin1 in chondrocytes induced by the HT-2 toxin was increased 0.88 fold after an incubation of 48 h. The increased expression level of Beclin1 in chondrocytes induced by the T-2 toxin was significantly higher when compared with that of the HT-2 toxin (24 h and 48 h), and the difference was statistically significant (*p* < 0.05).

## 3. Discussion

Currently, research efforts have focused mainly on T-2 toxin contamination in grains for the etiology of KBD. However, it remains unclear that whether the T-2 toxin in grains specifically damages articular cartilage of children KBD, and that the expression levels of apoptotic and autophagic proteins in chondrocytes are exposed to the T-2 toxin during the early stages of the disease. Based on our previous experiments [9,10], the T-2 toxin is metabolized to the HT-2 toxin after entering into the skeletal system of rats. The T-2 toxin levels were observed to decrease in chondrocytes over a 48 h period concomitant with the significant increase in the concentration of the HT-2 toxin. In contrast, there is a paucity of data describing the toxicity of the HT-2 toxin on chondrocytes, and there is no comparative study describing the toxicity of the T-2 and HT-2 toxins toward chondrocytes. Therefore, in this study, chondrocytes were incubated with the same concentration of the T-2 and HT-2 toxins to explore the apoptotic and autophagic affects induced by these toxins.

In this study, chondrocytes were incubated with the T-2 and HT-2 toxins for 48 h. MTT analysis revealed that the toxicity of the toxins on chondrocytes is time-dependent. The T-2 and HT-2 toxins increased oxidative stress in chondrocytes significantly. Flow cytometry analysis showed that the T-2 toxin induced an increase from 3.26% to 49.67% in the apoptosis rate of chondrocytes, whereas the apoptosis rate of chondrocytes induced by the HT-2 toxin increased significantly from 3.82% to 36.28%. Immunofluorescence analysis also confirmed that the apoptosis rate of chondrocytes induced by the two toxins increased significantly. Western blot analysis showed that the relative expression levels of Bax, caspase-3, and caspase-9 in chondrocytes incubated with the T-2 and HT-2 toxins increased significantly. The oxidative stress level, apoptosis rate, and apoptosis-related proteins for chondrocytes induced by the T-2 toxin were significantly higher than those observed when these cells were incubated with the HT-2 toxin. Nonetheless, the oxidative stress of chondrocytes incubated with both toxins increased significantly, which caused a change to the mitochondrial membrane potential and mitochondrial membrane osmosis, release of mitochondrial pro-apoptotic protein Bax, and the subsequent release of cytochrome C related proteins (caspase-9 and caspase-3). All these factors eventually resulted in apoptosis of the chondrocytes.

Autophagy of the chondrocytes was induced by both toxins, and immunofluorescence analysis showed that the autophagy rate of chondrocytes induced by the T-2 toxin increased from 3.94% to 60.67%, whereas the autophagy rate of chondrocytes induced by the HT-2 toxin increased from 3.74% to 40.75%. Western blot analysis revealed that the expression levels of Beclin1 increased significantly in chondrocytes incubated with the T-2 and HT-2 toxins. Autophagy of chondrocytes induced by the T-2 toxin was also significantly higher than that induced by the HT-2 toxin. Recent studies have reported that the T-2 and HT-2 toxins induce autophagy and apoptosis of porcine and mouse oocytes, rat brain, primary cardiomyocyte, liver cells, and mouse primary leydig cells. Two studies [11,12] showed an increase in the ROS levels of porcine and mouse oocytes when incubated with the HT-2 toxin, indicating an increase in oxidative stress. ROS levels in the treated group were also higher, confirming that the HT-2 toxin caused oxidative stress, which induced apoptosis and autophagy. A previous study [13] reported autophagy in the brain and apoptosis in the pituitary, suggesting that the T-2 toxin may induce different acute reactions in different tissues. Three studies [14,15,16] also confirmed that incubation of the T-2 toxin with mouse primary leydig cells, liver cells, and primary cardiomyocyte caused up-regulation of LC3-II and Beclin1, suggesting that the T-2 toxin promotes a high level of autophagy. Pretreatment of these cells with chloroquine and rapamycin was shown to increase and decrease the rate of apoptosis, respectively. Therefore, autophagy may prevent apoptosis of cells by reducing T-2 toxin-induced cytotoxicity. The T-2 toxin is also an environmental risk factor of the KBD, and our results showed that the T-2 and HT-2 toxins can significantly induce apoptosis and autophagy of chondrocytes, and these observations were consistent with previous studies [11,12,13,14,15,16,17]. The apoptosis and autophagy rates of chondrocytes induced by the T-2 toxin were much higher than those rates induced when the chondrocytes were incubated with the HT-2 toxin, and such an observation has not been reported previously.

The results showed an increase of both apoptosis and autophagy in chondrocytes treated with the T-2 and HT-2 toxins, which is in agreement with previous studies. Autophagy is a normal physiological activity of cells, which was activated in chondrocytes treated with the T-2 and HT-2 toxins, to avoid further cell damage. There is a complex relationship between autophagy and apoptosis. When cells are exposed to low environmental pressures, activation of autophagy can prevent apoptosis and subsequent cell death. When cells are subjected to strong or prolonged environmental stress, the process of autophagy consumes excessive levels of intracellular proteins or organelles, leading to cell survival failure that promotes programmed cell death [18,19,20].

## 4. Conclusions

In conclusion, our results showed that the T-2 and HT-2 toxins induce apoptosis and autophagy of chondrocytes, and that the level of oxidative stress plays an important role in autophagy activation. The activation of autophagy can reduce oxidative damage and therefore functions in protecting chondrocytes from apoptosis through capture, elimination, and degradation of damaged mitochondria.

## 5. Methods and Materials

### 5.1. Reagents and Antibodies

Fetal bovine serum (FBS), dimethyl sulfoxide (DMSO), and Hoechst 33258 were purchased from Sigma-Aldrich (St Louis, MO, USA). The T-2 and HT-2 toxins were purchased from J&K Chemical Ltd (Beijing, China). The thiazolyl blue tetrazolium bromide (MTT) was purchased from Amresco (Solon, OH, USA). The malondialdehyde (MDA) kit and the superoxide dismutase (SOD) kit were purchased from the Nanjing Jiancheng Bioengineering Institute (Nanjing, China). The Bicinchoninic Acid (BCA) Protein Assay kit was purchased from TianGen Biotech (Beijing, China). Anti-Bax, anti-Caspase 9, anti-Caspase 3, and anti-Beclin1 antibodies were purchased from Cell Signaling Technology, Inc. (Danvers, MA, USA).

### 5.2. Cell Culture and Treatment

The human chondrocytes cell line (C28/I2) was cultured in DMEM/F12 medium with 9% FBS at 37 °C and 5% CO_2_ in a humidified atmosphere. Once the chondrocytes had reached a steady state of the exponential growth phase, these cells were seeded at a density of 1.0 × 10^4^ per well in 96-well plates and grown overnight. The cells were then cultured in a medium containing either the T-2 toxin or the HT-2 toxin (20 ng/mL) for 0, 12, 24, and 48 h. The T-2 and HT-2 toxins (1 mg) were freshly dissolved in 1 mL DMSO and protected from light. In the T-2 toxin and HT-2 toxin treatment group, the cellular viability, oxidative stress, apoptosis, and autophagy of chondrocyte were determined.

### 5.3. MTT Assay

Human chondrocytes in the logarithmic phase were suspended in 0.1% EDTA trypsin. Two hundred microliter cell suspensions were seeded into individual 96-well plates at a density of 1 × 10^4^ cells per well. The experiments were carried out in the toxin group and control group. Complete medium with either the T-2 toxin or HT-2 toxin (20 ng/mL) was added and the cells were incubated for 0, 12, 24, and 48 h. Twenty microliters of MTT was added into the toxin and control groups to a final concentration of 0.5 mg/mL at each incubation time point. After 4 h at 37 °C, the medium containing MTT was aspirated and replaced with 150 μL DMSO and incubated for a further 1 h. Following this incubation, the absorbance was measured using an automatic microplate reader at 510 nm. The calculation of the viability rate at different concentrations and time points is as follows (1):

Viability rate (%) = {[(control group − blank control group) − (toxin group − blank toxin group)]/(control group − blank control group)} × 100%(1)

### 5.4. Oxidative Stress

Sample pretreatment: The supernatant of cell culture was discarded, and the pellet was digested with 0.25% trypsin for 2 min. Then, the culture medium was added to stop digestion by gentle micropipetting, and transferred into an EP tube and centrifuged at 3500–4000 rpm for 10 min. The supernatant was discarded and the precipitated cells were broken into suspension using ultrasonic wave. Their protein concentration was determined using bicinchoninic acid (BCA) protein assay kit. A volume of 0.2 mL of the suspension in a centrifuge tube (1.5 mL) was used for the assay.

The MDA assay kit was purchased from Nanjing Jiancheng Bioengineering Institute and used to measure oxidative stress damage. The centrifuge tubes were divided into four groups: standard tubes (0.2 mL 10 nmon/mL standards + 0.2 mL reagent 1 + 1.5 mL reagent 2 + 1.5 mL reagent 3), standard blank tubes (0.2 mL absolute ethyl alcohol + 0.2 mL reagent 1 + 1.5 mL reagent 2 + 1.5 mL reagent 3), measure tubes (0.2 mL measure sample + 0.2 mL reagent 1 + 1.5 mL reagent 2 + 1.5 mL reagent 3), and measure blank tubes (0.2 mL measure sample + 0.2 mL reagent 1 + 1.5 mL reagent 2 + 1.5 mL 50% glacial acetic acid). A spiral vortex mixer was used to mix samples in the standard tubes, standard blank tubes, measure tubes, and measure blank tubes. Test tubes were placed in a water bath for 40 min at 95 °C, cooled with a water cooling tube, and centrifuged at 3500–4000 rpm for 10 min. The supernatant was collected and the absorbance value (OD) of samples in each tube was measured at 532 nm with 1 cm optical path (2).

MDA (nmol/mgprot) = [(OD measure tube − OD measure blank tube)/(OD standard tube − OD standard blank tube)] × concentration of standard sample (10 nmol/mL) ÷ protein concentration of measure sample (mgprot/mL)(2)

The SOD assay kit was also purchased from Nanjing Jiancheng Bioengineering Institute and used to determine the activity of SOD using the WST-1 method. The centrifuge tubes were divided into four groups: control tubes (20 μL double distilled water + 20 μL enzyme working solution + 200 μL substrate application solution), control blank tubes (20 μL double distilled water + 20 μL enzyme diluents solution + 200 μL substrate application solution), measure tubes (20 μL measure sample + 20 μL enzyme working solution + 200 μL substrate application solution), and measure blank tubes (20 μL measure sample + 20 μL enzyme diluents solution + 200 μL substrate application solution). A spiral vortex mixer was used to mix samples in the control tubes, control blank tubes, measuring tubes, and measuring blank tubes, and then samples were incubated at 37 °C for 20 min. The absorbance value (OD) of samples was measured at 450 nm. The SOD activity is then measured by the degree of inhibition of this reaction. One unit of SOD was defined as the amount of enzyme needed to produce 50% dismutation of superoxide radical. The calculation of SOD activity is as below (3):

(U/mgprot) = inhibition rate of SOD (%) ÷ 50% × [reaction system (0.24 mL)/dilution ratio (0.02 mL)] ÷ protein concentration of measuring sample (mgprot/mL)(3)

### 5.5. Flow Cytometry of AV/PI

The apoptosis assay kit was used to measure apoptosis using Annexin V and PI double staining. Chondrocytes were incubated with either the T-2 or HT-2 toxin (20 ng/mL) for 0, 12, 24, and 48 h. Chondrocytes were washed with PBS twice and 250 μL binding buffer was added to resuspend chondrocytes at a density of 1.0 × 10^6^/mL. The cell suspension (100 μL), PI solution (10 mL, 20 μg/mL), and Annexin V/FITC (5 μL) were added to the 5 mL flow tube. The flow tube was mixed and incubated for 15 min in the dark at room temperature. Then 400 μL PBS was added to the reaction tube for flow cytometry analysis. As Annexin V and PI double staining were used to measure the apoptosis rate, automatic compensation regulation was used to avoid overlapping of two fluorescein wavelengths in the flow cytometry. When obtaining data from flow cytometric analysis, the gating was set using the combination of Forward Scatter (FSC) and Side Scatter (SSC), to establish FSC versus SSC dot diagram. By setting FSC threshold according to the size and granularity of chondrocytes, it can distinguish different cell populations, and remove from cell fragments, dead cells, and adhesion cells. The early apoptotic cells had been quantified using the Gated% data, the calculation of early apoptosis rate was Lower Right/(Upper Left + Upper Right + Lower Left + Lower Right).

### 5.6. Fluorescence Intensity Analysis

Hoechst 33258 was used to detect apoptosis of the chondrocytes. Chondrocytes were cultured in a 12-well plate. After the cells absorbed to the plate, the supernatant was discarded and the chondrocytes were fixed with 4% formaldehyde for 30 min. The fixed chondrocytes were stained using a Hoechst 33258 working solution for 1 h at 37 °C with 5% CO_2_ in a humidified atmosphere. The maximum excitation and emission wavelength of Hoechst-DNA were 352 and 461 nm, respectively. Nuclei of normal chondrocytes fluoresced blue under the fluorescence microscope, whereas pale, dense, and hyperchromatic nuclei represented apoptotic cells.

Monodansylcadaverine (MDC) can be used to specifically mark the formation of autophagosomes. The chondrocytes were treated with the T-2 and HT-2 toxins in 24-well plates, and the medium was absorbed. One hundred microliters of the MDC staining solution was added to each well and staining was carried out for 30 min at room temperature in the presence of light. The culture medium was discarded, and the cells were washed three times with 1× wash buffer (300 μL). The cell slide was covered with the collection buffer (100 μL). The wavelengths of stimulation and blocking filters of the fluorescence microscope were of 355 and 512 nm, respectively. Cell nuclei stained cyan-green were positive for an acidic autophagosome. When the cells were photographed using the fluorescence microscope, we identified four microscope fields of every replication microscopic picture (×400) and the cells were counted. Then the apoptosis rate was calculated as number of apoptotic cells / number of apoptotic cells and normal cells. Finally, the results were presented by mean ± standard deviations.

### 5.7. Protein Extraction and Western Blot Analysis

The chondrocytes were lysed using RIPA (Trizol method) and total protein in cell lysates was harvested by centrifugal separation according to the manufacturer’s instructions. The concentration of extracted protein was quantified by the BCA assay kit (Beijing Tiangen Biotech Company, Beijing, China). Equal amounts (50 μg) of extracted protein were subjected to 10% (w/v) sodium dodecyl sulfate-polyacrylamide gel electrophoresis (SDS-PAGE) and electrophoretically transferred onto PVDF membrane. They were pre-incubated in blocking buffer containing 5% (w/v) non-fat milk with Tween 20 for 60 min at room temperature, rinsed three times with TBST for 5 min. The membranes were incubated with different primary antibodies against Bax, caspase-9, caspase-3, and Beclin1 (Cell Signaling Technology, Boston, MA, USA) overnight at 4 °C; all primary antibodies were used at a 1:1000 dilution. After being washed three times in TBS, the membrane was incubated with an appropriately diluted horseradish peroxidase-labeled secondary antibody (1:5000) in blotting buffer for 30 min. The blots were visualized by enhanced chemiluminescent (ECL). Western blot signals were exposed to X-ray films and the bands were quantified by Quantity One software. The protein levels were standardized by comparison with anti-GAPDH antibody.

### 5.8. Statistical Analysis

All experiments were performed in three independent trials, each of which included three replications. Experimental data were presented as the mean and standard deviations. SPSS18.0 software (IBM, Armonk, NY, USA) was used to analyze the experimental data, and the *t*-test was used to compare the differences between two groups. *p* < 0.05 was considered to be statistically significant between two groups.
